# Application of contact-resonance AFM methods to polymer samples

**DOI:** 10.3762/bjnano.11.154

**Published:** 2020-11-12

**Authors:** Sebastian Friedrich, Brunero Cappella

**Affiliations:** 1 Federal Institute for Material Research and Testing (BAM), Unter den Eichen 87, 12205 Berlin, Germany

**Keywords:** atomic force microscopy, contact resonance, mechanical properties, polymers, wear

## Abstract

Contact-resonance AFM (CR-AFM) has been used in recent years for the measurement of mechanical properties of rather stiff materials, such as ceramics or metals, but also of some polymers. Compared with other techniques providing information on the mechanical properties of a sample, notably force–distance curves, CR-AFM has a much shorter acquisition time. This compensates in part the incomplete theoretical understanding of the underlying physical phenomena and of factors influencing the measurements. A commonly used method to analyze CR data requires the determination of the relative position of the tip, the calculation of the normalized contact stiffness, and the use of a calibration sample for the calculation of the elastic modulus of the sample. In the present paper, we propose an alternative procedure, based on approximations of the equations describing the system, which allows one to determine the elastic modulus of the sample as a parameter of the fit of the CR frequency as a function of the load. After showing that CR modes including scanning under continuous contact wear and damage the sample and/or alter the surface roughness, the results of point CR measurements on bulk and thin films are presented. Though Young’s moduli of bulk polystyrene and poly(methyl methacrylate) could be determined through the presented analysis, it is concluded that CR measurements are not appropriate for polymer samples. Major drawbacks are the bad resolution for moduli lower than ca. 10 GPa and the lack of a comprehensive physical model accounting for many factors affecting the dynamic response of a cantilever in contact with a sample.

## Introduction

The development of new materials for applications on the nanoscale, such as thin polymer films, demands a reliable determination of their mechanical properties. Atomic force microscopy (AFM) is a very versatile tool in surface characterization and provides, besides its initial intention of topographical imaging, several methods for obtaining information about mechanical properties. The most common and reliable method consists in recording force–distance curves, which is a quasi-static method. A thorough theoretical understanding of force–distance curves has been developed over the years, which enables a quantitative analysis of the elastic moduli for many kinds of systems [1–3]. The major drawback of force–distance curves is their long acquisition time. A force volume, which consists of a large number of curves in well-defined spatial intervals, can take several hours to complete. In order to overcome these drawbacks, dynamic scanning methods are a promising alternative to force–distance curves. For example, intermittent-contact (or tapping) mode AFM shows sensitivity to mechanical properties in the phase image. The resulting contrast is, however, hard to analyze quantitatively.

Contact-resonance AFM (CR-AFM) [4–5] is a dynamic contact technique that makes use of the vibrational behavior of the cantilever while the tip is in permanent contact with the sample. Generally, an increase in sample stiffness prompts an increase of the contact-resonance frequency (CR frequency). The CR frequency can be obtained from single-point measurements or tracked during scanning with techniques such as dual AC resonance tracking (DART) [6–7]. The vibrational motion of the cantilever is usually described using the Euler–Bernoulli beam equation [8–10]. In the simplest model, only the vertical elastic forces are represented by a spring between tip and sample. This can be extended by a spring in lateral direction as well as dashpots connected in parallel to the spring to account for viscoelastic interactions. Such models allow one to calculate the contact stiffness from the measured CR frequency. Then, from the contact stiffness, the elastic modulus of the sample can be determined. This technique has been successfully applied on rather stiff materials such as silicon [11] or chalcogenide glasses [12], as well as on some polymeric materials [13–15].

A requirement of the analysis based on the Euler–Bernoulli beam equation is that certain properties and dimensions of the probe are known. A crucial parameter is the location of the tip on the cantilever, which strongly influences the calculation of the contact stiffness. Several methods to obtain this parameter, such as direct measurement via scanning electron microscopy [14] or identification of the value for which two different modes of the same cantilever yield the same contact stiffness (“mode crossing” method), may lead to different values [16]. This is a major weakness of current analysis methods. Additionally, as in other techniques dealing with mechanical properties, the radius of the tip needs to be known. Alternatively, a calibration sample can be used [16]. Yet, this presupposes the exact measurement of the elastic modulus of the calibration sample. These requirements have prompted several alternative theoretical approaches [17–19], in some cases based on finite element analysis [13,20–21], as well as modifications of the measurement procedure [22].

In addition to the aforementioned critical points in quantitative analysis, scanning CR-AFM modes such as DART are affected by problems such as sudden jumps in the recorded CR frequency, which are probably caused by the collection of dirt particles by the tip during scanning [13]. This means that not only quantities calculated from the CR frequency, for example, Young’s modulus, but also the measured CR frequency itself are affected by large uncertainties and are often not reproducible. Therefore, several CR-AFM studies on polymers are limited to the mere detection of contrasts in CR frequency, without further calculations and, hence, without a quantitative determination of stiffness and moduli [20,23–25].

To compete with the well-established force-distance curves method in determining elastic properties of samples, CR-AFM must be able to produce reliable, reproducible values for CR frequencies. Additionally, an analysis method is necessary that does not rely on imprecise parameters such as the tip position on the cantilever.

This article shows quantitative CR measurements on polymer films of polystyrene (PS), poly(methyl methacrylate) (PMMA), and poly(*n*-butyl methacrylate) (PnBMA), as well as glass. Current analysis methods are simplified to a point that CR frequencies can be directly fitted to estimate the elastic modulus, without calculation of the sample stiffness and without the use of a calibration sample. Advantages and limitations of CR techniques are elucidated, with focus on polymer samples.

## Theory

The central goal of contact-resonance AFM (CR-AFM) is to get information on the stiffness of a sample via its vibrations and, in particular, through its contact-resonance frequency (CR frequency). In the following, the cantilever is modeled as a rectangular, elastically isotropic beam of uniform cross section with length *L*, width *w*, thickness *b*, density ρ, and Young’s modulus *E*_t_. The tip mass, being typically much smaller than the cantilever mass, is neglected. The tip is located at a distance *L*_1_ < *L* from the clamped end of the cantilever. The flexural spring constant of the cantilever is 
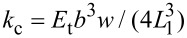
 [2].

The tip–sample interaction can be modeled by a vertical and a horizontal spring and a dashpot accounting for dissipative forces [16,26]. Yet, these sophisticated models lead to rather complex equations with a large number of parameters. In the simplest model, the tip–sample interaction is completely elastic and along a direction normal to the sample surface. The system can be represented by a spring with elastic constant *k*_s_, that is, the contact stiffness. Taking advantage of the Euler–Bernoulli beam equation, the normalized contact stiffness α = *k*_s_/*k*_c_ is given by [8–10,27]:

[1]
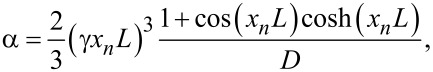


where γ is the relative position of the tip, given by *L*_1_/*L*, and *D* is given by:

[2]
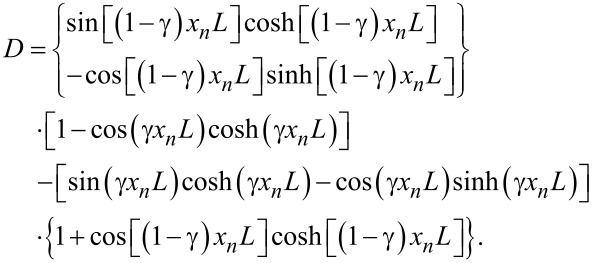


The wavenumber *x_n_L* for the contact flexural mode *n* can be calculated from 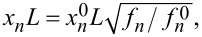 where *f_n_* and *f_n_*^0^ are the CR frequency and the free resonance frequency for the *n*-th flexural mode, respectively.

The wavenumber 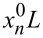 for the free flexural mode *n* can be calculated from the following equation describing the vibration of a cantilever of length *L* in free space [16,27]:

[3]
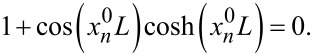


The first two roots of Equation 3 are 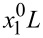 = 1.8751 and 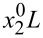 = 4.6941 [28].

Equation 1 has two important features affecting the feasibility of CR measurements. First, it can be used only to calculate the normalized contact stiffness α as a function of *x_n_L* or *f_n_*, but, since the equation cannot be inverted analytically, *x_n_L*(α) and *f_n_*(α) can be calculated only numerically. This also means that, when expressing the stiffness as a function of the static load *F* via a suitable elastic continuum theory [3], such as Hertz theory [29], only the function *F*(*f_n_*) can be determined analytically, and not its inverse *f_n_*(*F*).

Second, the parameter γ is usually determined through a procedure (“mode crossing”, see [16]), which is not always feasible [10,27,30], “may differ slightly from the physical tip position” [16], and may even vary for different measurements with the same cantilever (see the Results section).

In the following, we will approximate and simplify Equation 1 with two aims: (1) to reduce it to an equation that can be inverted, thus allowing the analytical calculation of *f_n_*(α) or else *f_n_*(*F*); and (2) to better understand the meaning of the parameter γ.

The first approximation is based on the fact that γ is usually in the range 0.9 ≤ γ ≤ 1, hence (1 − γ)*x_n_L* ≪ 1. It is known that, for ε ≪ 1, sin ε ≅ sinh ε ≅ ε and cos ε ≅ cosh ε ≅ 1. Substituting in Equation 2, we get:

[4]
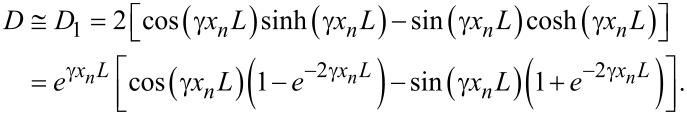


For the second approximation, we assume that γ*x_n_L* ≫ 1 and exp(−2γ*x_n_L*) ≅ 0. Hence:

[5]
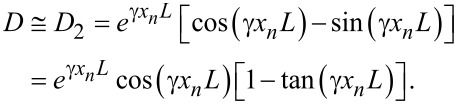


In the following, the function *D* and its approximations are discussed for the first mode, that is, for 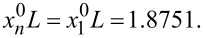 Except for numerical results, all equations are valid also for higher modes, provided the corresponding value of 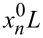 is used.

**Figure 1:**
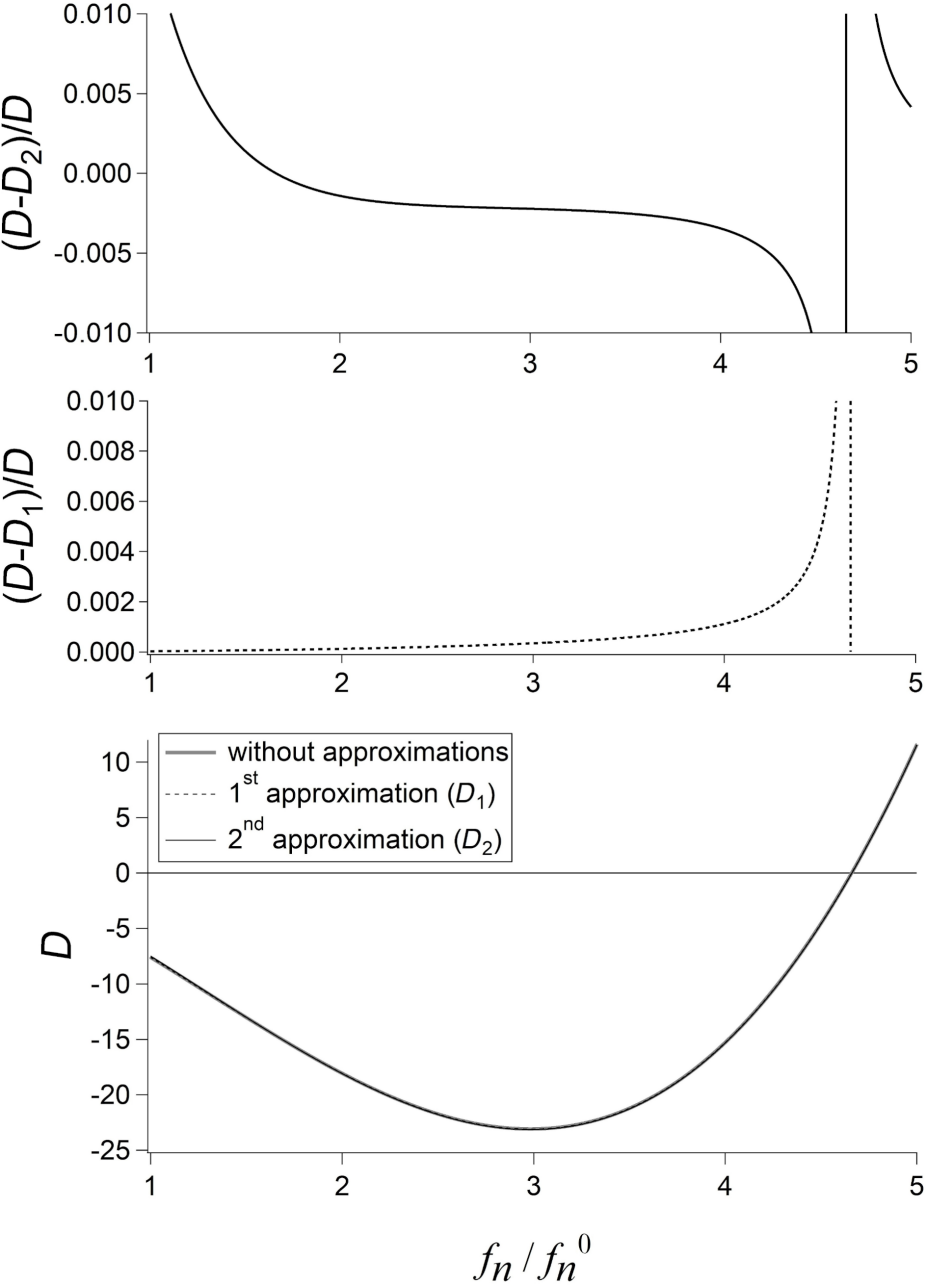
The function *D* without approximations (Equation 2) and with both approximations (Equation 4 and Equation 5) for γ = 0.97. The top part of the figure shows the differences between both approximated functions and the exact one, divided by the exact function.

Figure 1 shows *D* without approximations (Equation 2) and with both approximations (Equation 4 and Equation 5) for γ = 0.97 and 1 ≤ *f_n_*/*f_n_*^0^ ≤ 5. The top part of the figure shows the differences between both approximated functions and the exact one, divided by the exact function. Both approximations are very good. The three curves can hardly be distinguished from each other. The first approximated function is always smaller than the exact one and the normalized difference exceeds 1% only next to the root of *D*. For the second approximated function, the normalized difference is higher than 1% only next to the root of *D* or for *f_n_*/*f_n_*^0^ ≅ 1.

The second approximation given by Equation 5 allows one to calculate analytically the root of *D*, that is, the value (*f_n_*)_0_ at which α diverges. It is given by (*x_n_L*)_0_ = 5π/4γ, since (*x_n_L*)_0_ = π/4γ would yield the unphysical result *f_n_*/*f_n_*^0^ < 1. For the first mode, we get for the root of *D*:

[6] $${\left(f_{n}\right)}_{0}=f_{n}^{0}{\left(\frac{1}{1.8751}\frac{5}{4}\pi \frac{1}{\gamma }\right)}^{2}.$$

With γ = 0.97 and (*f_n_*)_0_ = 4.66152*f_n_*^0^, the numerical solution is (*f_n_*)_0_ = 4.65985*f_n_*^0^. Hence, the deviation is 0.036%. Even for γ = 0.9, the deviation between the root given by Equation 5 and the numerical solution is smaller than 0.8%. Yet, even this small discrepancy plays an important role in the exact determination of γ and can be overcome through an alternative expression of *D*:

[7]
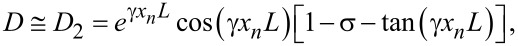


with an ad hoc parameter σ, which can be calculated from higher terms in the approximations of sin[(1 − γ)*x_n_L*], sinh[(1 − γ)*x_n_L*], cos[(1 − γ)*x_n_L*], and cosh[(1 − γ)*x_n_L*] and is a function of γ.

With Equation 7, the divergency of the normalized difference (*D* − *D*_2_)/*D* at (*f_n_*)_0_ = 4.65985*f_n_*^0^ almost disappears (<6 × 10^−3^) and, for 2*f_n_*^0^ < *f_n_* < 4.65*f_n_*^0^, is −2 × 10^−4^ < (*D* − *D*_2_)/*D* < −8 × 10^−4^.

Turning to the contact stiffness α, it is important to remember that the numerator of Equation 1 is negative for values of *x_n_L* between 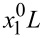 = 1.8751 and 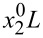 = 4.6941, that is, 1 < *f*_1_/*f*_1_^0^ < 6.267. Since the function *D* is negative for 0 < *f*_1_/*f*_1_^0^ < 4.66, the maximum attainable CR frequency in the first mode is *f*_1_ ≅ 4.66*f*_1_^0^, because larger frequencies would lead to an unphysical negative contact stiffness.

The second approximation, applied to α, yields:

[8]
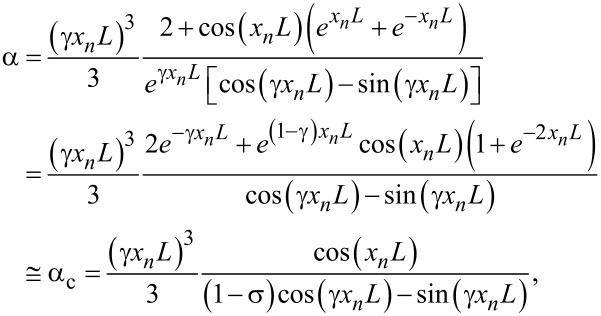


with the same σ as in Equation 7. Even if considerably simplified, Equation 8 still cannot be inverted. Further simplifications are necessary. Using simple trigonometric relations, Equation 8 can be written as

[9]
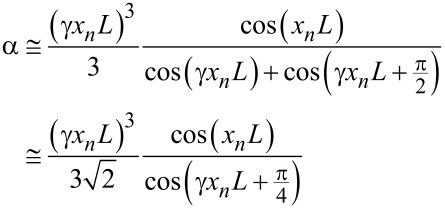


and, using again sin ε ≅ ε≅ 0 and cos ε ≅ 1 for ε ≪ 1, we finally get:

[10]
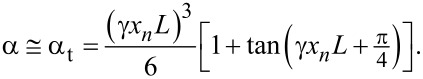


Since CR frequencies are usually more than three times higher than free resonance frequencies, the function on the right-hand side of Equation 10 can be developed in Taylor series around 5π/4. By putting γ*x_n_L* − 5π/4 = ξ, we get:

[11] $$\alpha ≅\frac{1}{6}{\left(\xi +\frac{5}{4}\pi \right)}^{3}\left[\frac{-1+\sum_{1}^{\infty }\frac{{\left(-1\right)}^{n+1}}{\left(2n\right)!}\xi^{2n}}{\xi +\sum_{1}^{\infty }\frac{{\left(-1\right)}^{n}}{\left(2n+1\right)!}\xi^{2n+1}}+1\right].$$

Around 5π/4, the most important term is the one that diverges, that is, the one proportional to 1/ξ. Therefore, we can approximate α as follows:

[12]
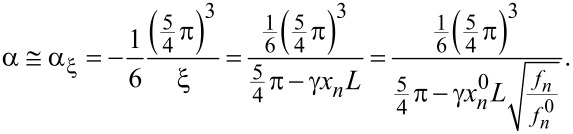


The approximated function α_ξ_ diverges at *x_n_L* = 5π/4γ. Again, an ad hoc parameter can be employed to eliminate the discrepancy of the divergency points.

**Figure 2:**
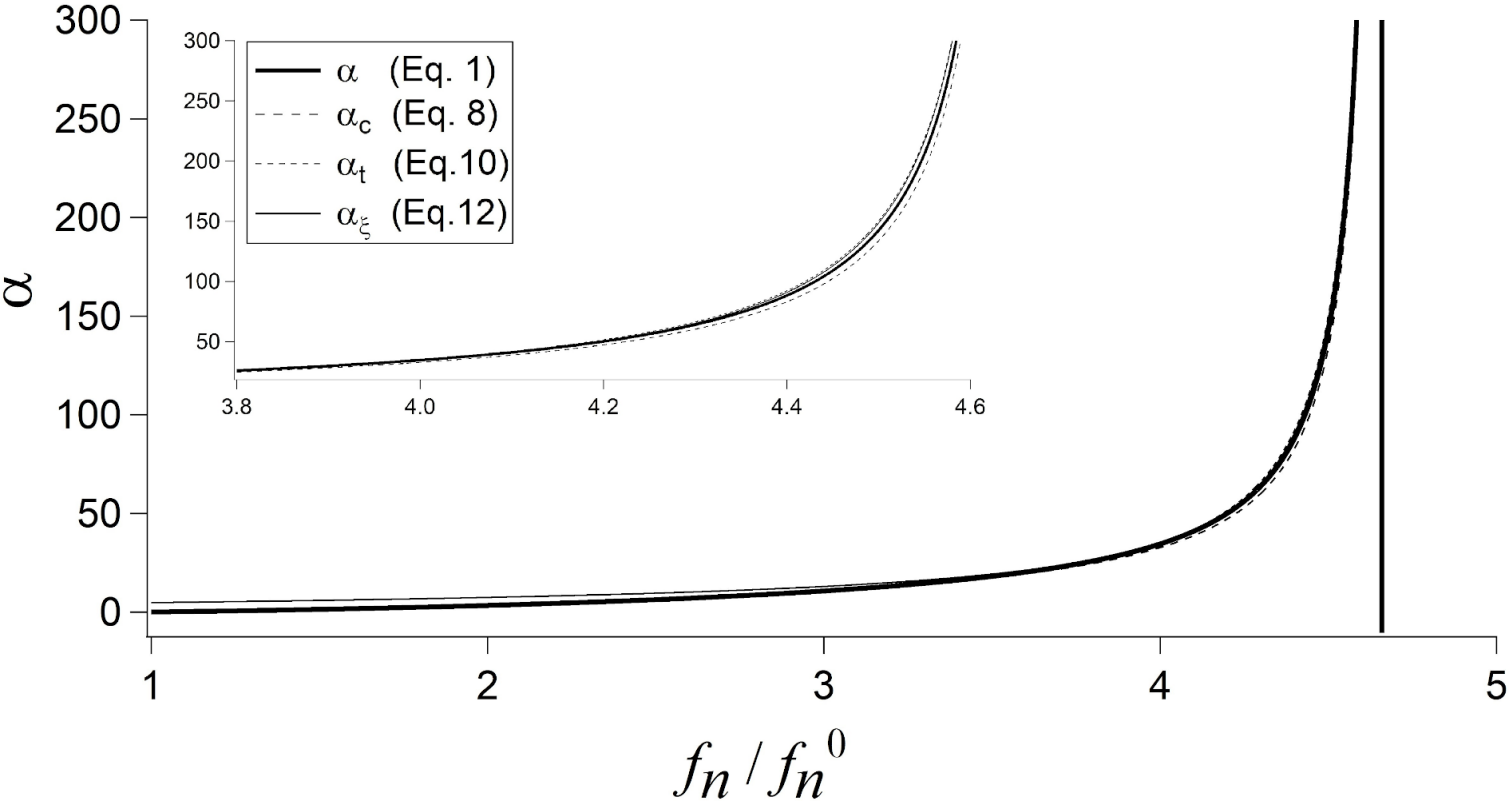
The function α without approximations (Equation 1) and its three approximations α_c_ (Equation 8), α_t_ (Equation 10), and α_ξ_ (Equation 12). The inset shows in detail the range 3.8 < *f_n_*/*f_n_*^0^ < 4.6.

Figure 2 shows the function α without approximations (Equation 1) and its three approximations, that is, α_c_ (Equation 8), α_t_ (Equation 10), and α_ξ_ (Equation 12), as a function of the frequency ratio *f_n_*/*f_n_*^0^. We can roughly distinguish three ranges: *f_n_*/*f_n_*^0^ < 3.5, where α is almost constant, a transition range for 3.5 < *f_n_*/*f_n_*^0^ < 4.3, and 4.3 < *f_n_*/*f_n_*^0^ < (*f_n_*)_0_, where α rapidly increases and finally diverges.

The best approximation is clearly α_t_, whereas α_c_ and α_ξ_ differ substantially from α in the transition range and for small frequencies (*f_n_*/*f_n_*^0^ < 3.5 in Figure 2), respectively. Yet, as already noticed, CR frequencies are usually more than three times higher than free ones. Moreover, the discrepancy between α and α_ξ_ is counterbalanced by the fact that α_ξ_ can be inverted and, hence, can be used to calculate *f_n_*(α) analytically.

Since Equation 12 can be inverted analytically, it is useful to express the contact stiffness as a function of the static load *F*. For a spherical or a paraboloidal tip and for a homogeneous sample, if sample adhesion to the AFM tip is negligible, that is, if sample deformation can be described through Hertz theory [1,3,29], the sample stiffness is given by:

[13]
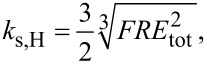


where *R* is the tip radius and the reduced elastic modulus *E*_tot_ is obtained through:

[14]
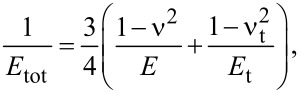


with *E* and *E*_t_ and ν and ν_t_ denoting the moduli and Poisson’s ratios of sample and AFM tip, respectively. Expressions for other solids of revolution are well known ([3], page 13).

Since α_H_ = *k*_s,H_/*k*_c_, combining Equation 12 and Equation 13 we get:

[15]
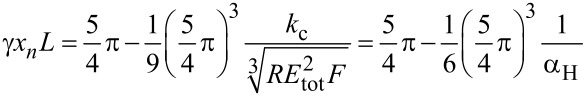


and finally

[16]
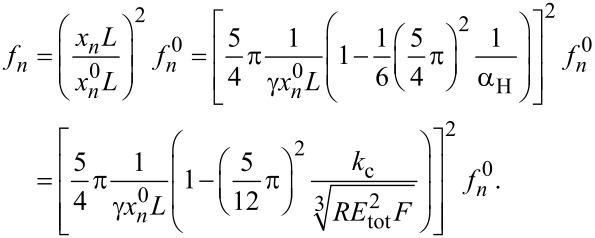


This equation allows one to fit the curve *f_n_*(*F*), that is, the CR frequency measured at different loads. Provided the radius of the tip and, of course, the elastic constant of the cantilever are known, the fit function can be used to estimate the relative position of the tip γ and, more important, the elastic modulus *E* of a sample the deformation of which can be described through Hertz theory. For α_H_ < 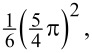 Equation 16 would yield negative values of 
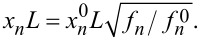
 This unphysical result restricts the range of application of Equation 16 to

[17]
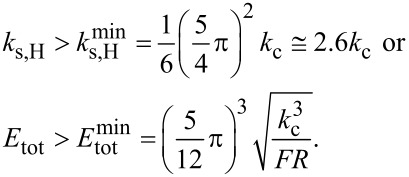


This limit in modulus for the application of Equation 16 is no practical restriction. Even with a very sharp tip (*R* = 5 nm), *F* = 50 nN, and *k*_c_ = 3 N/m, 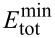 = 0.7 GPa and Equation 16 can be applied to measurements on several polymers having a modulus larger than 1 GPa, such as PS or PMMA.

Before testing Equation 16 in experiments, two of its properties are worth being highlighted. First, for α_H_ ≫ 1, that is, for 
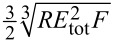
 = *k*_s_ ≫ *k*_c_, *f_n_* tends to

[18]
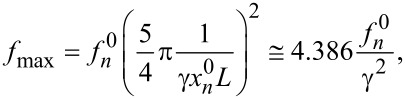


where the numerical approximation has been calculated for the first mode, that is, 
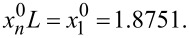
 This confirms that, for a given cantilever, the CR frequency has an upper limit. Furthermore, measurements with very large forces or tip radii or on very stiff samples can be used to estimate the parameter γ without “mode crossing”. Whereas the parameter γ determines the asymptotic value of *f_n_*, the fitting parameter *E*_tot_ affects mainly the “width” of the derivative ∂*f*_n_/∂*F*, that is, “how fast” the asymptotic value is attained. Hence, the two parameters are indeed independent.

Second, the first derivative of the CR frequency with respect to the elastic modulus is:

[19]
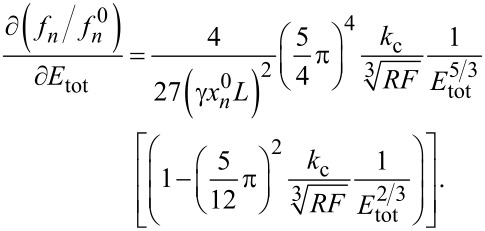


For 
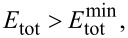
 the function in Equation 19 is monotonically decreasing and tends to 0 for *E*_tot_→∞. Furthermore, the term in square brackets can be neglected in Equation 19. Hence, the first derivative of the CR frequency is proportional to 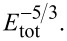 This means that, at small values of the elastic modulus, a small change of *E*_tot_ engenders a large change of *f_n_*, whereas, at large values of the elastic modulus, the contact resonance is less sensitive for changes in the elastic modulus of the sample. In other words, for a certain kind of cantilever, that is, when the elastic constant and the tip radius do not vary, the resolution of the measurement is less good with stiff samples than with compliant ones. It is interesting to determine the error Δ*E*_tot_ engendered by an uncertainty Δ*f_n_*/*f_n_*^0^ = 1%, corresponding, for most cantilevers, to Δ*f_n_* = 1–4 kHz:

[20]
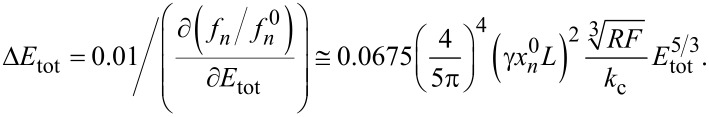


**Figure 3:**
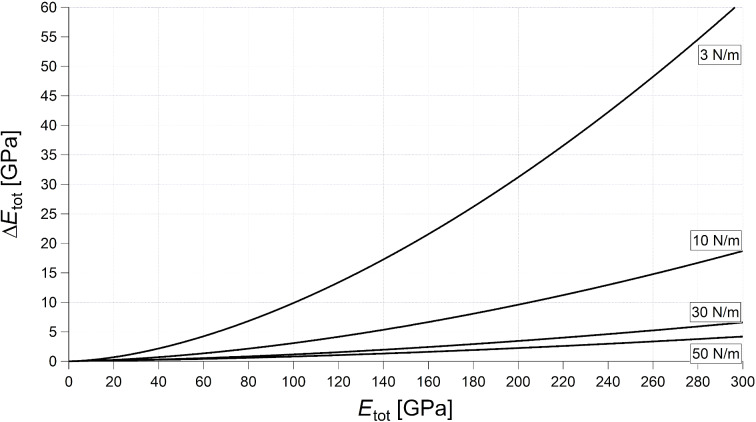
Error Δ*E*_tot_ engendered by an uncertainty Δ*f_n_*/*f_n_*^0^ = 1%, calculated from Equation 20 with γ = 0.97, *F* = 100 nN, *R* = 30 nm, and four values of *k*_c_ (3, 10, 30, and 50 N/m), as indicated by the labels.

Figure 3 shows Δ*E*_tot_ for γ = 0.97, *F* = 100 nN, *R* = 30 nm, and four values of *k*_c_ (3, 10, 30, and 50 N/m). As expected, Δ*E*_tot_ increases with *E*_tot_. For *k*_c_ = 3 N/m, it is Δ*E*_tot_ ≅ 22 GPa at *E*_tot_ ≅ 160 GPa (14%), Δ*E*_tot_ ≅ 4.2 GPa at *E*_tot_ ≅ 60 GPa (7%), and Δ*E*_tot_ ≅ 0.03 GPa at *E*_tot_ ≅ 3 GPa (1%). The error Δ*E*_tot_ is inversely proportional to *k*_c_ and decreases substantially with stiffer cantilevers. Yet, stiffer cantilevers are less suited for measurements on polymer samples, since they are likely to damage the sample. Moreover, the limit given by Equation 17 increases with *k*_c_ and the model proposed in this article could not be applied. Acquiring frequencies at smaller forces improves the resolution, but just slightly, because the error is proportional to 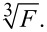 For example, in measurements with a force *F* = 10 nN, which is very small and hardly feasible, the error would decrease only by a factor of two. As suggested by Rabe et al. [9], also higher modes can be used to improve the resolution. This is not in contradiction to Equation 20. Δ*E*_tot_ is proportional to 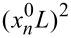 and 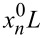 increases with the mode number, but *f_n_* increases too, so that Δ*f_n_* = 1–4 kHz corresponds to less than 1% of *f_n_*. Therefore, the factor 0.01 in Equation 20 is considerably lower and compensates the increase of 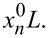 Due to the increase of CR frequencies with the mode, measurements at higher modes are not always feasible, since microscopes have usually an upper limit of detectable frequencies (2 MHz for a standard Cypher setup). Also, the amplitude of the oscillation, that is, the height of the resonance peak, decreases with increasing mode. As a consequence, for most cantilevers, measurements in modes higher than the fourth are not feasible.

It is instructive to compare the error Δ*E*_tot_ of a CR measurement with the corresponding error of a force–distance curve measurement. To this aim, the system is modelled by a spring of “constant” *k*_s,H_ (Equation 13), depending on the force. The elastic modulus is calculated from the measured value of δ/*Z*, where δ is the cantilever deflection and *Z* the piezo displacement ([3], page 9). The error given by 1% uncertainty in the measurement of δ/*Z*, corresponding to a realistic uncertainty of 1 nm for a 100 nm contact line, is:

[21]
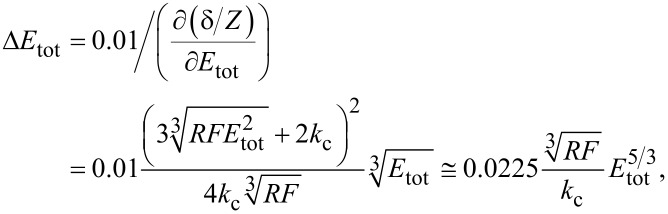


where the approximation is valid for 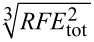 ≫ *k*_c_, that is, for very stiff samples.

Comparing Equation 20 and Equation 21, it is found that the error of a measurement of force–distance curves on very stiff samples (*k*_s_ ≫ *k*_c_) is approximately 25 times higher. Indeed, with *k*_c_ = 3 N/m, Δ*E*_tot_ > 10% already at 1 GPa and, even with *k*_c_ = 50 N/m, the error at 160 GPa is 22%.

Even this simplistic analysis shows that CR measurements are indeed more suitable than force–distance curves for the measurement of high moduli. Yet, measurements on such samples are feasible only with stiff cantilevers, which are not appropriate for measurements on compliant samples such as polymers, as will be shown later. This is a serious drawback when characterizing composite samples with large differences in the moduli of the components.

## Results

In order to test Equation 16, in a first approach, measurements in DART mode have been performed on several polymer samples with different cantilevers. The evident result of these measurements is that the polymer sample is worn or, more general, damaged during the scan. Scanning with the AFM tip leads to different forms of wear and modifications. When abrasion or plastic deformation are the dominant mechanisms, the DART measurement leads to a depression of the whole scanned area, eventually accompanied by the formation of pile-ups.

For example, measurements on a 120 nm thick PS film with a static force higher than ca. 250 nN leave depressions that are visible even with an optical microscope, and the depth of which increases with increasing static force (see Supporting Information File 1, Figure S1). Such plastic deformations of the polymer film engender changes of its mechanical properties, since compression leads to an increase of the stiffness. Moreover, if mobile polymer chains are pushed aside, as it is the case for PnBMA, the layer thickness is reduced, also leading to an increase of the stiffness and to severe changes of the sample [31]. In extreme cases, when the polymer film is completely worn, the AFM tip is in contact with the blank substrate and measures its properties, instead of those of the polymer film.

At lower static forces, ripples [32–34] may be formed. This well-known wear phenomenon has been studied in detail, mostly through scans in contact mode, that is, without oscillations of the AFM tip. In particular, it is known that the amplitude and the wavelength of the ripples increase with decreasing scanning speed [35] and with increasing load, temperature, or number of scans [35–36]. Some works have shown that oscillations of the AFM tip with increasing amplitude lead to a reduction of the ripples and finally to their suppression [37].

**Figure 4:**
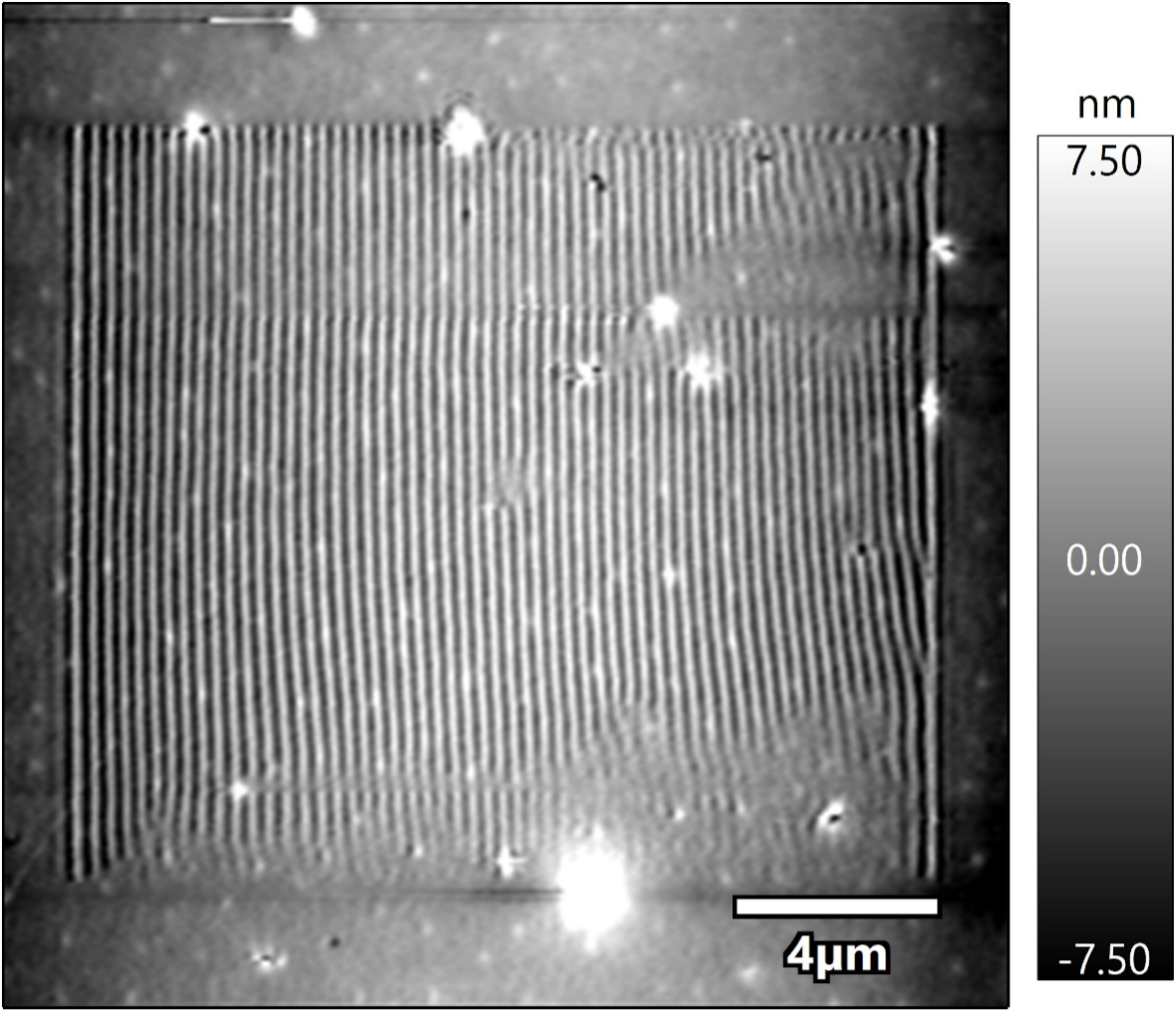
Tapping-mode topography image of a 100 nm thick PnBMA film after a smaller area (15 µm)^2^ was scanned in DART mode with a static force of 308 nN, a frequency of ca. 320 kHz, and amplitudes of 440 and 80 pm.

As an example, Figure 4 shows a tapping-mode topography image of a 100 nm thick PnBMA film scanned with a PPP-FMAuD cantilever (*k*_c_ = 2.74 N/m). The wave pattern was “engraved” into a smaller scan area of (15 µm)^2^ in DART mode previous to the scan in tapping mode. For the DART scan a static force of 308 nN, a frequency of ca. 320 kHz, and amplitudes of 440 and 80 pm were employed.

Some studies show that polymer samples on which ripples have been formed are more compliant than the corresponding unmodified samples [38]. This agrees with the hypothesis that ripples result from the formation of (microscale) cracks and voids in the polymer bulk. Though such results must still be confirmed by further studies, it is evident that ripples change the roughness of the sample, the geometry of the contacting surfaces and, hence, the contact area, which plays a crucial role in the characterization of mechanical properties.

**Figure 5:**
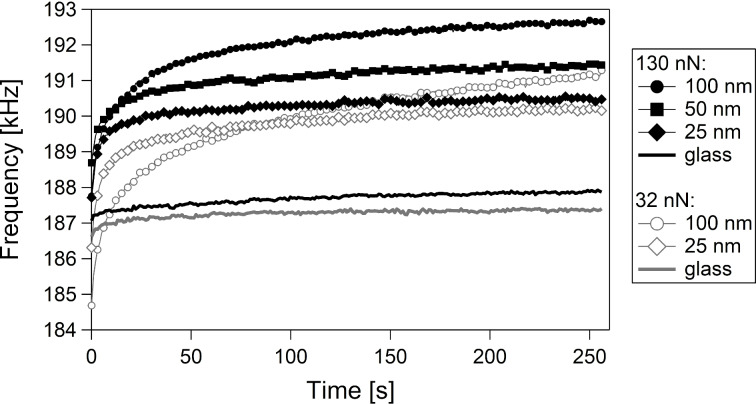
CR frequency *f*(*t*) in the first mode as a function of the measuring time on three PnBMA films with thickness of 25, 50, and 100 nm at a static force of 130 nN (full markers) and 32 nN (hollow markers, only 100 and 25 nm thick films). Measurements on glass at both 32 and 130 nN are shown as black and grey lines without markers. Frequencies have been averaged over the 128 points in one scan line, yielding one averaged value per second. For clarity, only one point out of four is shown.

Even when ripples are not formed and the static load is not high enough to induce a uniform depression, abrasion and plastic deformation can lead to the formation of disordered agglomerates of polymer chains, severely changing the roughness of the surface. Since these wear phenomena are due to the lateral movement of the tip, a second group of measurements has been performed. In this case, the lateral scan size was 1 nm, hence the tip does not move laterally. This allows for the characterization of the temporal evolution of the resonance frequency. An AC240 tip with *k*_c_ = 0.775 N/m was used. The static force was varied, too. Figure 5 shows the CR frequency *f*(*t*) in the first mode as a function of the measuring time on three PnBMA films with a thickness of 25, 50, and 100 nm at a static force of 130 nN (full markers) and 32 nN (hollow markers, only 100 and 25 nm thickness, because the 50 nm curve would make the graphic too unclear). Measurements on glass for both static forces are also shown (black and grey lines without markers). The resonance frequencies have been averaged over the 128 points in one scan line. With a scanning frequency of 1 Hz, this results in one averaged value per second, but, for clarity, only one point out of four is shown. On glass, *f*(*t*) increases rather little (by less than 1 kHz), but clearly, with time. This is due to moderate deformations of the sample and/or of the tip, increasing the contact area. On PnBMA, when the tip dwells on the sample, *f*(*t*) increases rather fast in the first few seconds, and then more slowly. The tip compresses the PnBMA film over time, thereby making it stiffer and increasing the CR frequency. The tip might also pierce through the PnBMA film and come closer to the substrate or even in direct contact with it. After a few seconds, frequencies on PnBMA exceed even the frequencies on glass, although glass is much stiffer. This occurs most probably because the plastically deformed PnBMA material surrounds the tip. Hence, a larger portion of the tip is in contact with the sample. In other words, the contact radius and consequently the sample stiffness (Equation 13) increase.

This is confirmed by the comparison of the different curves on PnBMA: (1) At 130 nN, the thicker the film, the higher the CR frequency. This counterintuitive result (thicker films are less stiff [31]) can be explained only through the fact that more material surrounds the tip on thicker samples. (2) At both static forces, the curves on the thinner films attain a plateau, whereas, on the 100 nm thick film, *f*(*t*) increases further after some minutes. This is because the thicker polymer film can be pierced deeper and the contact area increases further. (3) At 32 nN, for *t* smaller than ca. 80 s, *f*(*t*) on the 100 nm film is smaller than on the 25 nm thick film. This is due to the shielding effect of the thicker polymer film, preventing the tip from “sensing” the substrate and making the sample less stiff [31]. Yet, after ca. 80 s, a larger amount of polymer surrounds the tip and the contact area increases, so that *f*(*t*) on the 100 nm film becomes larger than on the 25 nm film. (4) Since the deformation of the sample and the contact area correlate with the static force, at a given time, *f*(*t*) at 130 nN is larger than at 32 nN on all samples.

**Figure 6:**
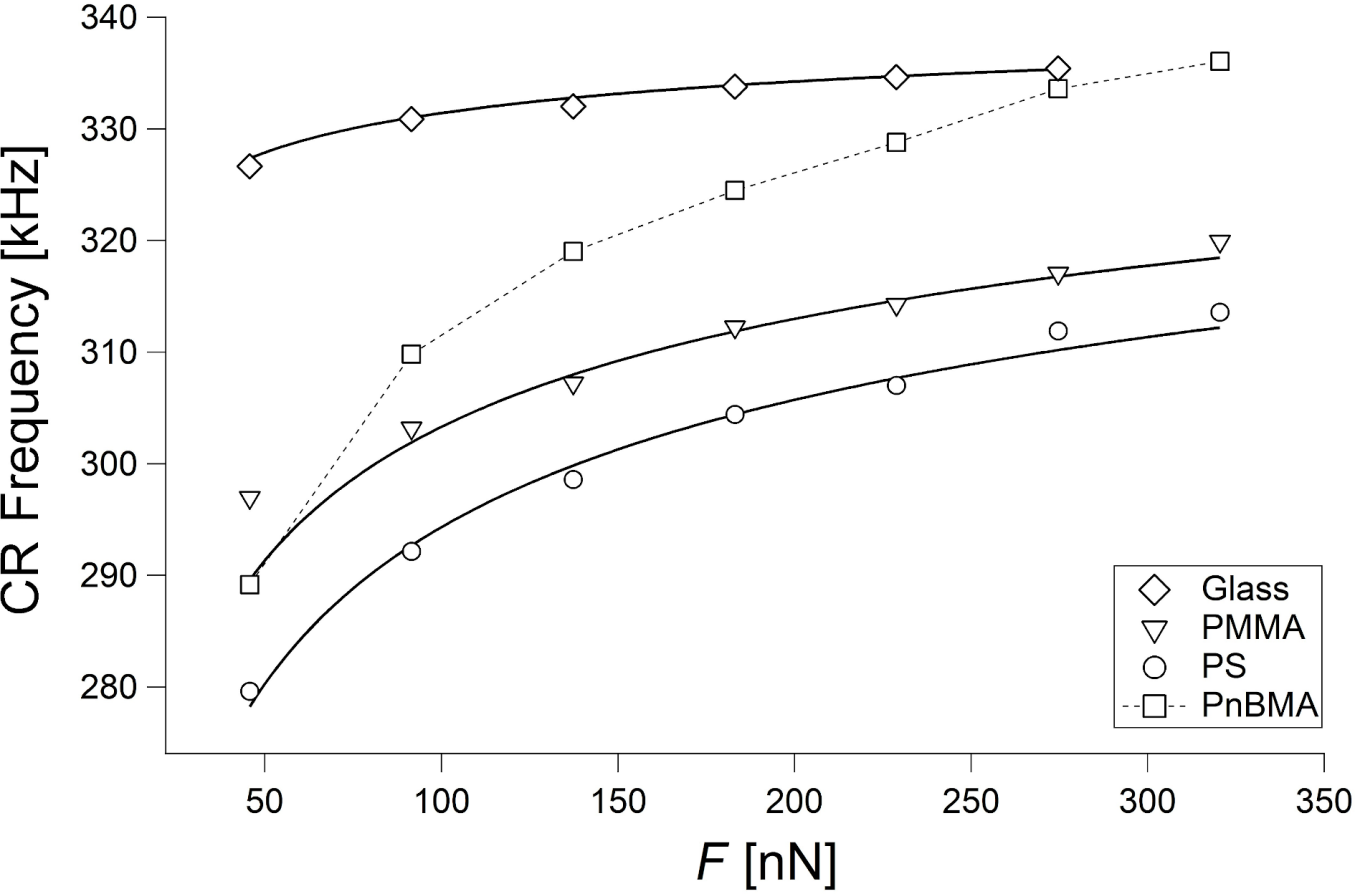
CR frequencies as a function of the force *F* on glass (diamonds), bulk PMMA (triangles), and bulk PS (circles), fitted with Equation 16 (black curves). CR frequencies on PnBMA (squares) are shown, too.

Since measurements with a lateral movement of the tip or even with the tip dwelling on the sample modify the sample and change its roughness and/or its mechanical properties, the experimental test of Equation 16 has been performed by means of point measurements of the CR frequency by variation of the force. Each measurement has been performed at a different position on the sample. The standard deviation of the six measurements performed at each force is typically between 0.1 and 1.5 kHz. Figure 6 shows the CR frequency in the first mode of a PPP-FMAuD cantilever as a function of the force *F* on glass, bulk PS and bulk PMMA, fitted with Equation 16, where the reduced elastic moduli *E*_tot_ and the relative tip position γ are the fit parameters. The parameters of the measurement are *f*_1_^0^ = 75.9 kHz, *R* = 55 nm, and *k*_c_ = 3.27 N/m. Hence, since *F* is larger than 50 nN, Equation 16 can be applied for *E*_tot_ > 0.3 GPa (see Equation 17). For all following measurements, the tip radius *R* was measured through recording tapping mode topography images on a grid with sharp tips (see Experimental section).

The curves can be fitted quite exactly, most of all those on glass and PS. The three values of the reduced elastic moduli obtained for glass, PMMA, and PS are 62.3 GPa, 9.4 GPa and 7.2 GPa, respectively; with *E*_t_ = 160 GPa, ν_t_ = 0.28 and a Poisson's ratio of 0.27 for glass, 0.4 for PMMA, and 0.33 for PS, the elastic moduli are 57 GPa, 6.2 GPa and 5 GPa, respectively. The measured values of the elastic moduli of PMMA and PS are higher than the literature values for the respective bulk polymers ([3], page 98). These higher values are due to inner stresses and the stretching of the polymer chains during spin coating [3,31,39]. Furthermore, since CR measurements are performed at high frequencies, the time–temperature superposition principle leads to a stiffening of the sample [40]. This effect, while being rather moderate for polymers having a glass transition temperature *T*_g_ higher than room temperature, such as PMMA and PS, strongly affects the thermomechanical properties of polymers with a *T*_g_ comparable with or lower than room temperature. The measured value of the elastic modulus of PS is in agreement with previous measurements on spin-coated samples [3]. The value obtained on bulk PMMA has been checked through force–distance curves on the sample. The measurement performed with a 40 nm tip yielded a reduced elastic modulus of 9.5 GPa, in agreement with the CR value (see Supporting Information File 1, Figure S2). The second fitting parameter, γ, is different for the three curves (0.982 for glass and 0.973 for PS and PMMA), although they have been acquired with the same cantilever. This contradicts the interpretation of γ as determined only by the relative position of the tip.

Figure 6 also shows the CR frequencies acquired on bulk PnBMA. The curve cannot be fitted with Equation 16. This is due to the low elastic modulus and the higher chain mobility of PnBMA. As a consequence, PnBMA is very compliant and can be deformed plastically even with small loads. Hence, at loads of approximately 300 nN, even during a short measurement, PnBMA has been displaced laterally, that is, the tip has carved a hole in the polymer film and is in contact with a very thin PnBMA film or even with the glass substrate. Therefore, the CR frequency goes from typical “polymer values” around 290 kHz at 50 nN to typical “glass values” around 330 kHz at 300 nN.

**Figure 7:**
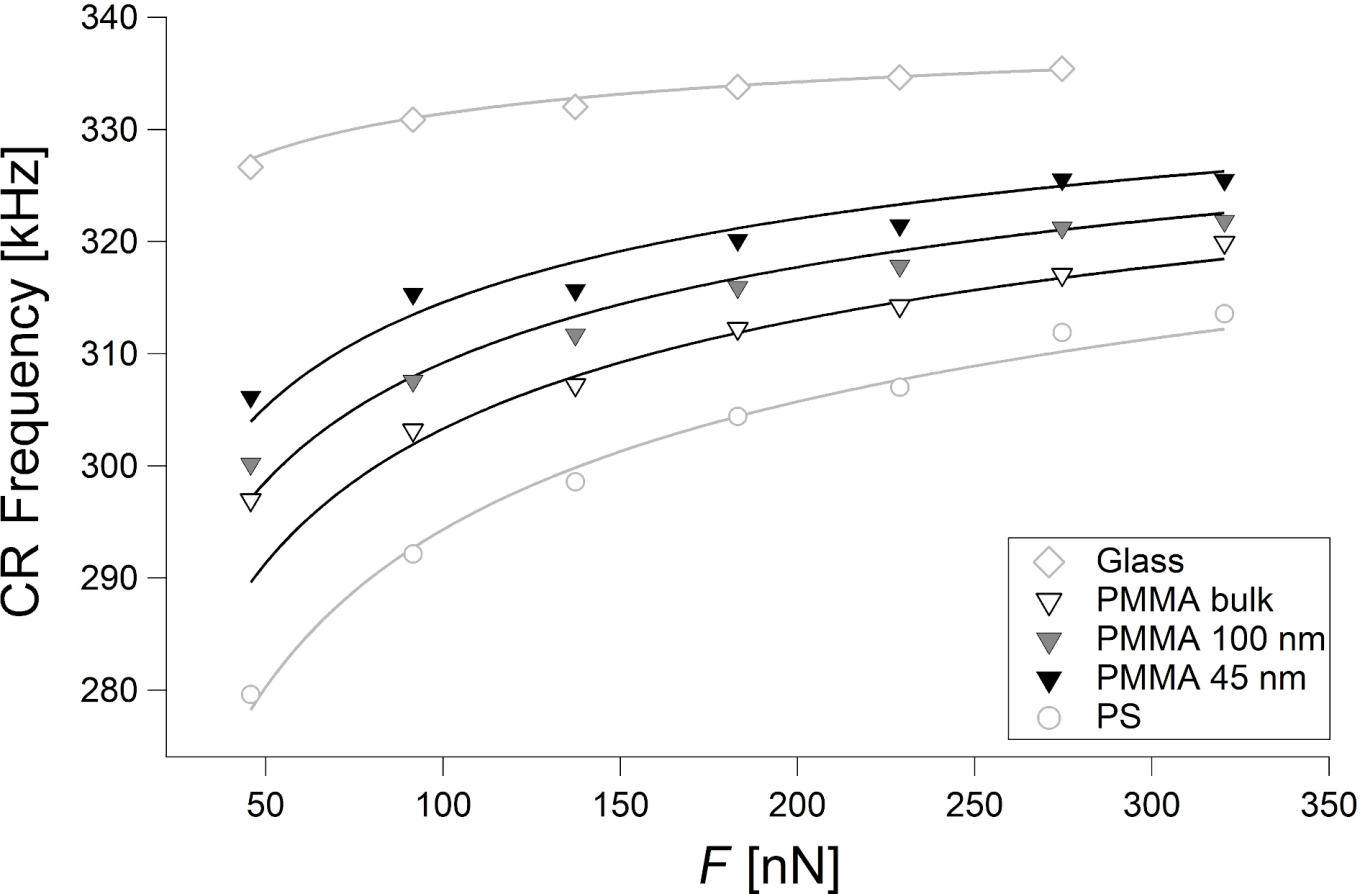
CR frequency as a function of the force *F* on glass (diamonds), bulk PMMA (white triangles), and bulk PS (circles), as in Figure 6. CR frequencies on two PMMA films, 45 nm and 100 nm thick are shown, too (grey and black triangles, respectively). All curves are fitted with Equation 16 (light grey and black curves).

Figure 7 shows again the CR frequencies on glass, bulk PS and bulk PMMA, together with CR frequencies on two PMMA films with thickness values of 45 ± 5 nm and 100 ± 2 nm, measured with the same cantilever. The two additional curves on the thin PMMA films are fitted with Equation 16, too. The parameter γ is the same as for bulk PMMA and bulk PS (0.973).

The CR frequencies on thin PMMA films depend on the thickness via the elastic modulus [41]. The reduced elastic modulus is *E*_tot_ = 11.5 GPa (*E* = 7.6 GPa) for the 100 nm thick film and *E*_tot_ = 14.2 GPa (*E* = 9.5 GPa) for the 45 nm thick film. Hence, CR measurements enable to distinguish films of different thickness with moduli differing by 1–2 GPa. However, the thickness resolution is clearly worse than that of force–distance curves. A comparison with [41] shows that force–distance curves enable the clear distinction of six different thickness values between a 45 nm thick film and bulk PMMA, whereas data scattering and the small differences between the frequencies (≅4 kHz) would hardly allow one to discern a further curve in Figure 7 between those corresponding to the 45 nm film and the 100 nm film or even between those of the 100 nm film and of the bulk sample.

**Figure 8:**
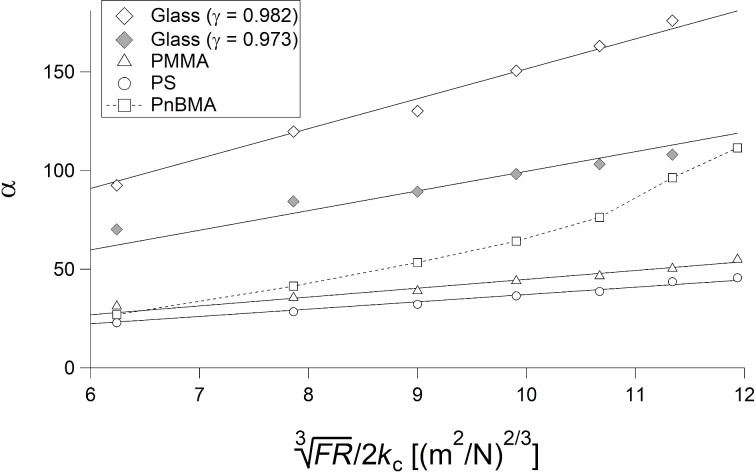
Normalized contact stiffness α on glass (white diamonds, calculated with γ = 0.982, and grey diamonds, calculated with γ = 0.973), bulk PMMA (triangles, γ = 0.973), and bulk PS (circles, γ = 0.973), fitted with a straight line through the origin (black lines). The normalized contact stiffness on PnBMA (squares, γ = 0.973) is shown, too.

The values of γ determined through the fit have been used to calculate α (Equation 1). Figure 8 shows α as a function of 
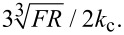
 In such a plot, if the deformation of the sample can be described by Hertz theory, α can be fitted with a straight line through the origin; the slope of the line is given by 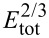 (see Equation 13).

The normalized contact stiffness on glass (white diamonds, γ = 0.982), bulk PMMA (triangles, γ = 0.973), and bulk PS (circles, γ = 0.973) can indeed be fitted with a straight line through the origin. When using γ = 0.973 for glass (grey diamonds), the fit is not as good as for γ = 0.982; the experimental data clearly have a smaller slope than the fit, but a straight line with this slope would not go through the origin. Furthermore, this fit yields a reduced modulus *E*_tot_ = 31 GPa, which is definitely too low for glass. As shown in this example, the proportionality between α and 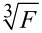 can be used to test values of γ, provided measurements are performed on homogeneous samples that are deformed only elastically and exhibit small adhesion, that is, the deformation of which can be described by Hertz theory. The normalized contact stiffness on PnBMA (squares, γ = 0.973), as expected, is not proportional to 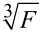.

**Figure 9:**
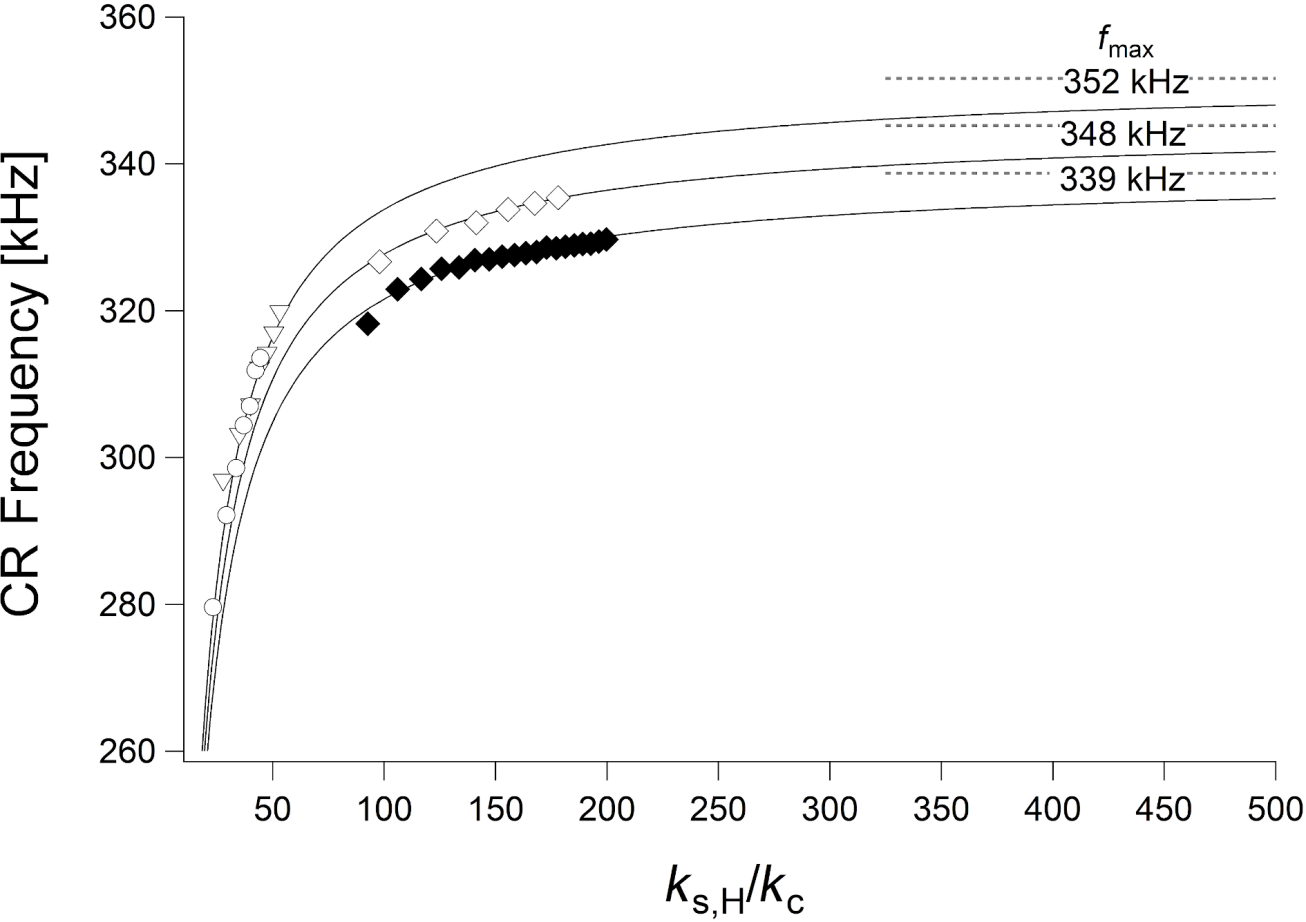
CR frequency as a function of the normalized contact stiffness α_H_ = *k*_s,H_/*k*_c_ on glass (open and full diamonds, two different measurements), bulk PMMA (triangles), and bulk PS (circles), fitted with Equation 16 (black curves). Dashed grey lines denote the maximun CR frequency attainable with the the respective values of *f*_1_^0^ and γ.

Figure 9 shows the CR frequencies of the previous measurement (open diamonds for glass, triangles for bulk PMMA, and circles for bulk PS) together with an additional measurement on glass (full diamonds), performed with a PPP-FMAuD cantilever (*R* = 55 nm, and *k*_c_ = 3.27 N/m) with a free resonance frequency of *f*_1_^0^ = 75.85 kHz. Frequencies are plotted as functions of the normalized contact stiffness α_H_ = *k*_s,H_/*k*_c_. Hence, they would lie on the same curve, if the value of γ was the same for all samples. The fit of the additional measurement with Equation 16 yields γ = 0.991 and *E*_tot_ = 65.3 GPa, corresponding to *E* = 63.2 GPa. The value of γ is considerably different from the two other ones. This, again, contradicts the interpretation of γ as the relative tip position.

The maximum CR frequency attainable with the respective values of *f*_1_^0^ and γ, that is, the frequency *f*_max_ given by Equation 18, is noted in Figure 9, too. It is evident that, in all three measurements, *f*_max_ has not been reached. Both measurements on PS and PMMA are in the range where the frequency rapidly increases with increasing stiffness, that is, with increasing force. However, even the highest CR frequency measured on the second glass sample (full diamonds) is 330 kHz, that is, significantly lower than *f*_max_ = 339 kHz. In practice, the static load cannot be raised indefinitely, since this would lead to wear of the tip, especially on hard and stiff samples such as glass.

## Discussion

The first tests performed in this work with a CR method (DART) on polymers show definitely that scanning CR measurements, that is, with the cantilever moving not only normally to the surface, but also laterally, are unable to provide reproducible quantitative results. The main reason for this is wear and damage of the sample while scanning in permanent contact. Summarizing, the following forms of wear or damage are observed: (1) At high loads, when abrasion is the dominating wear mechanism, the tip removes the polymer film on top of the substrate during the scanning. Debris is accumulated at the sides of the scan surface. If the removal is total, with progressing scan movement, the tip senses the substrate and not the polymer film. Even if the erosion is only partial, since the stiffness of a thin polymer films depends on its thickness [31], the measurement of mechanical properties is severely affected by the wear. (2) Another wear mechanism that, depending mainly on the cohesion and linking of the polymer chains, can take place at high loads as an alternative to abrasion is plastic wear. When this form of wear occurs, the scan leads to the formation of a depression, eventually accompanied by pile-ups at the sides of the scan surface. In this case, the mechanical properties are altered because of the changes in thickness and, more important, because of compression. (3) Even when the load is not high enough to modify the whole scan surface through abrasion or plastic deformation, local wear may lead to the formation of agglomerates, which increase the roughness of the sample. Via changes in the contact geometry and in the contact radius ([3], page 83), this phenomenon affects significantly the determination of mechanical properties. (4) At lower normal forces, ripples may be formed, height and distance of which depend, among others, on the thermomechanical properties of the polymer, on the load, the scanning speed, the number of scans, and the temperature. Even if the mechanism of ripple formation is not known exactly, it is reasonable to assume that the mechanical properties of a polymer sample with ripples are significantly different from those of an unmodified sample. In particular, polymer chains in the depressions are likely to be tightly packed or compressed, whereas those in the protrusions are probably looser and enclose air or water cavities. Even without supposing changes in the mechanical properties of the samples, the changes in the sample geometry and, hence, in the contact area affect their measurement.

Since CR frequencies are particularly sensitive to tip contamination, a further problem is that debris or wear particles, but also dirt or loose particles on the surface, are likely to adhere onto the tip. An additional experiment in CR mode with the tip dwelling for some minutes on the same point shows that, also in this case, the sample is modified, and its properties change during the measurement. Hence, the only way to detect the properties of polymer samples without altering them is to measure the CR frequency during a short contact.

A commonly used method to analyze CR data [9–10,16,26] includes the following steps: (1) the determination of the relative position of the tip, γ, through “mode crossing”, (2) the calculation of the normalized contact stiffness α with measured CR frequencies and the value of γ determined in the first step, and (3) the calculation of the elastic modulus of the sample through comparison with a calibration or reference sample, provided the shape of the tip is known.

The alternative method we have proposed in this work, based on an approximation of the equation describing α, enables to fit the CR frequencies measured at different loads directly, without determination of the relative position of the tip through mode crossing, without the previous calculation of the normalized contact stiffness α, and without recurring to a calibration sample. Yet, not only knowledge of the shape of the tip is necessary, but also of geometrical parameters (for example, the radius of the tip in case of spherical or paraboloidal shape). The fit yields values of the elastic moduli of glass, PMMA, and PS, which are in agreement with literature values obtained with force–distance curves. Moreover, differences in the elastic modulus due to film thickness can be detected (Figure 7).

The relative position of the tip, γ, is the second fit parameter. Previous works have shown that values of γ yielded by SEM measurements and calculated through mode crossing are not the same [10,26]. Differences are ascribed to deviations of the cantilever shape from the idealized model shape (uniform rectangular cross section). The measurements analyzed in the present work show varying γ values for the same cantilever. For example, it is γ = 0.973 for PS and PMMA, but γ = 0.982 (or γ = 0.991 in another measurement) for glass. Different values of γ for the same cantilever are obtained not only with the method proposed in this work, but also with mode crossing. In [9], it has been shown that curves α(γ) calculated for the first three modes do not intersect at the same point. This contradictory result is attributed to discrepancies between the model and the real experimental conditions. Nevertheless, there is actually no criterium to decide which value of γ should be used in the further analysis, and researchers should arbitrarily choose one intersection, knowing that this leads to errors of the modulus values of ca. 20% [9,26]. More important, the values obtained for γ may differ even when considering the same two modes but using different samples. In our experiments, the largest difference between the values of γ measured through mode crossing has been found during measurements on PS films of different thickness (1–1.3 µm and 120 nm), yielding γ = 0.966 and γ = 0.98, respectively. Hence, the parameter γ is not a measure of the relative tip position alone and depends also on the sample. In particular, values of γ determined on compliant polymer samples are often very different from those on stiff samples, such as glass or silicon. This is probably due to the use of very simple models: (1) The cantilever is modelled as an elastically isotropic beam of uniform cross section and the tip mass is neglected [8,26]. (2) The sample is described by Hertz theory, that is, plastic deformations, viscoelastic behavior, and adhesion are neglected [3,40]. Yet, adhesion has been shown to be indeed negligible for measurements on polymers such as PS and PMMA with a customary AFM tip [3]. (3) The description of the cantilever–sample system as a vertical spring ignores lateral forces (and related torsion) and damping [8,26].

The models do not represent satisfactorily the complex situation of a CR measurement on a polymer. In other words, different values of γ for different samples “compensate” for the lack of parameters accounting for other factors. In particular, it has been shown that the influence of lateral forces increases with *k*_s_ and that, when including them in the model, curves α(γ) corresponding to different modes intersect indeed at the same point [26]. However, accounting for anisotropies in the cantilever structure, tip mass, plastic deformations, viscoelastic behavior, adhesion, lateral forces, and damping increases significantly the number of free parameters, so that the practical use of such complex models is very limited.

The dependence of γ on the sample is a severe limitation for measurements on thin films of very compliant polymers (*E* < 1 GPa), the mechanical properties of which are determined by the mechanical properties of the polymer at small indentations and by the mechanical properties of the substrate at large indentations. The same limitation holds for measurements on very compliant polymers with high chain mobility, that is, with a glass transition temperature near room temperature. This is the case for PnBMA. Considering the CR frequencies (Figure 6), PnBMA behaves like PS and PMMA at small forces and like glass at large forces. In our measurements, γ does indeed depend on the sample, since using for glass the same value of γ obtained for PS and PMMA (γ = 0.973) yields a value of the elastic modulus that is too low, and the contact stiffness cannot be described by Hertz theory. Hence, the calculation of α is not possible for PnBMA. As a matter of fact, the CR frequencies and the contact stiffness for PnBMA in Figure 6 and Figure 8 would agree with each other only if the parameter γ used for PnBMA gradually increased from 0.973 to 0.982 with increasing load.

As already pointed out, the direct fit of CR frequencies without a reference or calibration sample for the calculation of moduli requires the knowledge of geometrical parameters, for example, the tip radius. This is a drawback, since measurements on test gratings may damage the tip and blunt it. Moreover, small deviations from the paraboloidal shape, for example, small protrusions, are likely to seriously affect deformations and the measurement of mechanical properties. Finally, the determination of the tip radius is rather erroneous for very sharp tips.

A common way to circumvent this problem is the use of colloidal probes, that is, spheres with a known radius in the micrometer range glued onto tipless cantilevers ([3], pages 74–75). Yet, CR measurements with a silicon colloidal probe (radius *R* = 1.5 µm) performed on glass and bulk PS and PMMA yielded results that could not be analyzed quantitively, neither with our approximation (Equation 16), nor with the “exact” model (Equation 1). In particular, even with a large variation of γ and even including the adhesion force, measured separately with force–distance curves, as an additional force, the sample stiffness *k*_s_ is never proportional to 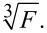 In case of colloidal probes, the cross section of the cantilever is not uniform, the mass of the “tip” is not negligible and the adhesion, at least on polymers, is comparable with the load. Hence, further developments in the theory are necessary for the quantitative use of colloidal probes.

## Conclusion

The measurements presented in this paper show that CR modes such as DART, performed with the tip in permanent contact with the sample, are likely to wear compliant polymer samples and/or to alter the sample surface, notably the roughness, and, hence, the contact area with the tip. Scanning CR methods are therefore not suitable for quantitative measurements of the elastic modulus on polymers.

Point measurements of the CR frequency have been shown to enable the accurate determination of the elastic modulus of glass, PS, and PMMA. Through approximation of the common equation describing the dynamic response of the cantilever as a function of the contact stiffness, CR frequencies as function of the applied load can be fitted directly. Yet, such experiments show also that quantitative measurements on polymer samples with elastic moduli smaller than ca. 1 GPa or with a glass transition temperature close to or lower than room temperature (i.e., with very high chain mobility) are affected by serious artefacts and do not yield any exact estimation of the moduli. Also, the characterization of the substrate influence on the mechanical properties of thin polymer films is not as detailed and fine as with force–distance curves. As the acquisition time of such point measurements is even longer than that of force curves, this method does not offer any advantages.

Like measurements through force–distance curves, such a quantitative analysis presupposes the knowledge of the tip radius and, of course, of the spring constant of the cantilever. Yet, a further parameter is needed with CR methods, namely the relative tip position γ. Differences in the values of γ yielded by different measurements with the same cantilever indicate that commonly employed models are not appropriate for polymer samples. Not only anisotropies in the cantilever structure, tip mass, and lateral forces, but also plastic deformations, viscoelastic behavior, adhesion, and damping should be accounted for in models of the system. Unfortunately, especially in case of polymers, this would drastically increase the number of parameters needed for the description of the cantilever–sample system. The inadequacy of simple models for the description of polymer samples is also shown by measurements with a colloidal probe, allowing for qualitative conclusions (e.g., comparison of the CR frequency on glass, PMMA, and PS) but not for the quantitative determination of the moduli of samples.

A comparative analysis of the errors of the values of the moduli engendered by an uncertainty in the measured frequency (CR modes) or in the measured deflection (force–distance curves) shows that CR methods are more suitable than force–distance curves for the measurement of moduli larger than ca. 20 GPa. Bulk polymer samples and thin polymer films commonly have lower elastic moduli.

## Experimental

### Materials

Contact-resonance measurements have been performed on films of polystyrene (PS, average *M*_w_ ≅ 280,000), poly(methyl methacrylate) (PMMA, average *M*_w_ ≅ 120,000), and poly(*n*-butyl methacrylate) (PnBMA, average *M*_w_ ≅ 337,000). All polymers have been purchased from Sigma-Aldrich (St. Louis, USA). PnBMA has a glass transition temperature *T*_g_ of 15 °C, while the *T*_g_ value of PS and PMMA is above 100 °C. The polymers have been dissolved in toluene and then spin-coated on glass cover slips, previously cleaned and rinsed with toluene. By changing the polymer concentration, the obtained sample thickness could be varied. Films with a thickness larger than 400 nm are considered as bulk, since the substrate does not influence the mechanical properties of the sample [31].

### Contact-resonance mode AFM

Contact-resonance atomic force microscopy (CR-AFM) measurements have been performed with a Cypher AFM (Asylum Research, Oxford Instruments, Santa Barbara, USA). Two kinds of silicon AFM tips have been used: PPP-FMAuD (*k*_c_ ≅ 3 N/m) from Nanosensors (NanoWorld, Neuchatel, Switzerland) and AC240 (*k*_c_ = 0.775 N/m) from Asylum Research. Additional measurements have been done with a silica sphere (Sigma-Aldrich) with a radius of 1.5 µm that has been glued to a NSC15 tipless cantilever with *k*_c_ = 40 N/m (MikroMasch, Sofia, Bulgaria) using two-component epoxy UHU Endfest 300 (UHU, Bühl, Germany).

Before the measurement, the sensitivity of the cantilever has been calibrated by recording force curves on an uncompliant substrate, such as a silicon wafer. The spring constant could then be determined from the thermal noise spectrum [42]. Tip radii have been obtained through scanning a TGT1 test grating (NT-MDT Spectrum Instruments, Moscow, Russia) consisting of an array of sharp tips. The resulting image is a replica of the AFM tip [43–44]. An example of tip imaging is shown in Supporting Information File 1, Figure S3.

Single-point CR measurements have been conducted by performing a frequency sweep (tune), while the tip has been in contact with the sample, and identifying the contact-resonance frequency *f_n_*. The excitation is actuated through the sample, which is glued to a contact-resonance sample holder by means of a two-component epoxy. To avoid sample damage, every point measurement is done on a different spot on the sample. Each value is averaged over four to six separate measurements. In different series of measurements, the static force exerted on the sample by the tip has been varied. A sufficiently small excitation amplitude has been chosen for the frequency sweep, so that the vibration amplitude is always smaller than the static indentation of the sample and the tip always remains in contact. Additionally, the dual AC resonance tracking (DART) mode [6–7] has been employed. In this mode the cantilever is excited at two frequencies on either side of *f_n_*, which allows one to track *f_n_* while scanning the sample in contact.

## Supporting Information

File 1 Additional figures.
