# Supplementary material for: Application of contact-resonance AFM methods to polymer samples
Source: Beilstein J Nanotechnol. 2020 Nov 12;11:1714–27. doi: 10.3762/bjnano.11.154 (PMC7670117; doi:10.3762/bjnano.11.154)
Supplement: File 1 — Additional figures. [file Beilstein_J_Nanotechnol-11-1714-s001.pdf]

BEILSTEIN JOURNAL OF NANOTECHNOLOGY

Supporting Information

for

Application of contact-resonance AFM methods to polymer samples

Sebastian Friedrich and Brunero Cappella

Beilstein J. Nanotechnol. **2020,** *11,* 1714–1727. doi:10.3762/bjnano.11.154

Additional figures

License and Terms: This is a supporting information file under the terms of the Creative Commons Attribution License (https://creativecommons.org/licenses/by/4.0). Please note that the reuse, redistribution and reproduction in particular requires that the authors and source are credited.
The license is subject to the *Beilstein Journal of Nanotechnology* terms and conditions: (https://www.beilstein-journals.org/bjnano)

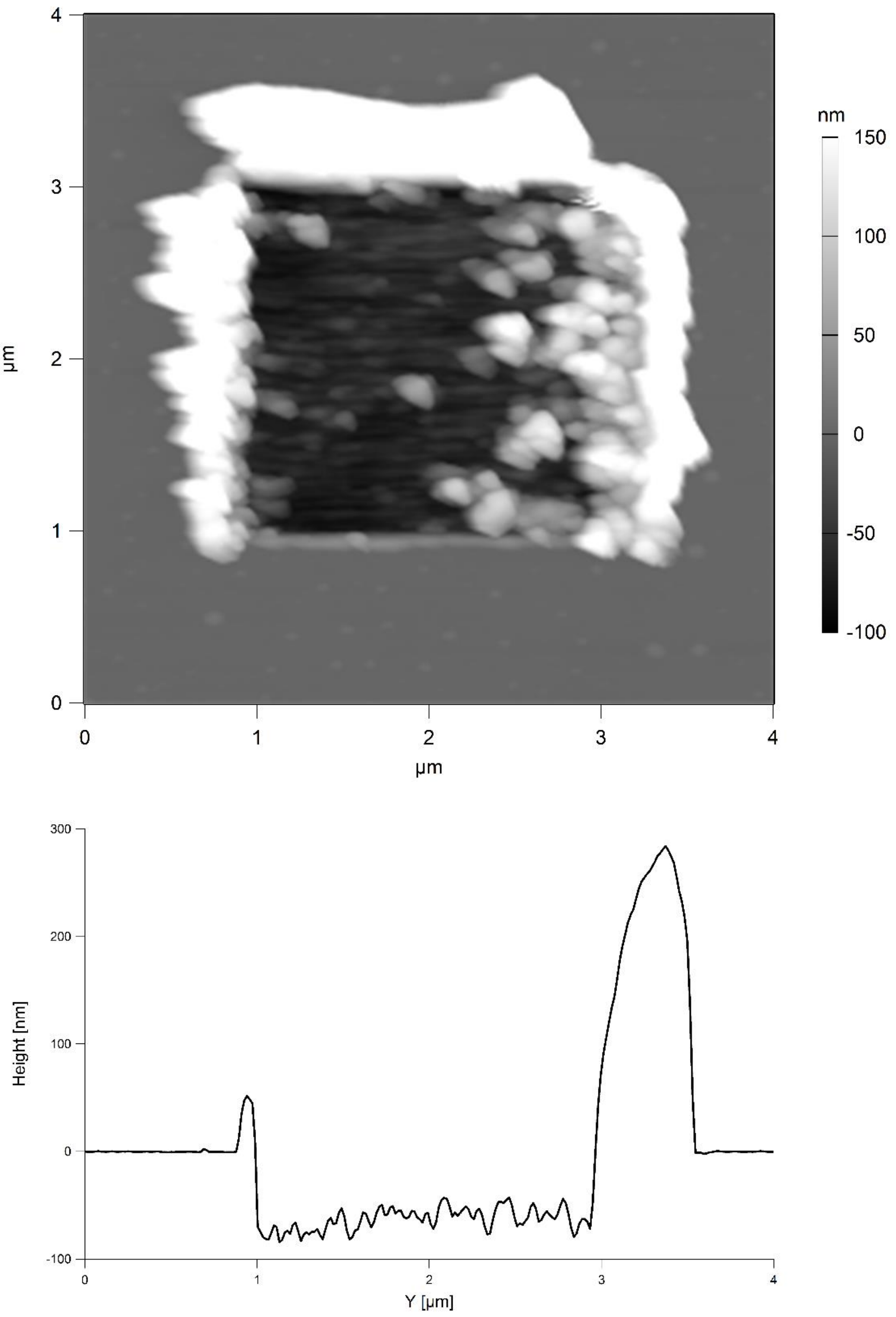

Figure S1: Wear of a PS film after scanning in DART Mode. Top: tapping-mode topography image of a 120 nm thick PS film scanned with a PPP-FMAuD cantilever (k_c = 5.58 N/m). The depression was carved into a smaller scan area of (2 µm)^2 in DART mode previous to the scan in tapping mode. For the DART scan a static force of 870 nN and a center frequency of ca. 360 kHz were used. An almost uniform depression with voluminous wear debris, most of all in the right part of the DART scanning area, is clearly visible. Bottom: line profile at X = 1.58 µm, showing a depression of 63 ± 10 nm.

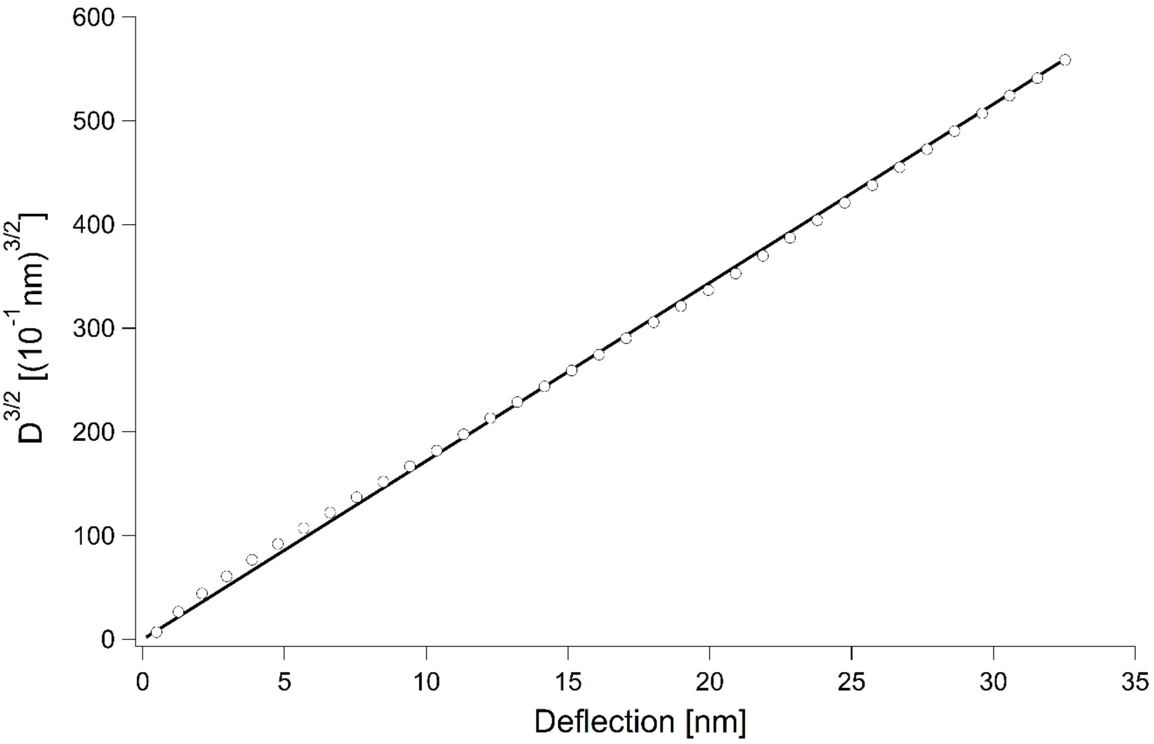

Figure S2: Fit of a D32-curve on PMMA. The deformation D of bulk PMMA was averaged from 100 force–distance curves as $D = Z - \delta$, where Z is the piezo displacement and δ the deflection of the cantilever. Curves were acquired with a cantilever with an elastic constant k_c = 32.8 N/m and with a tip radius R = 40 nm. Following Hertz theory, the deformation to the power of 3/2 as a function of the deflection is fitted with a straight line going through the origin. The reduced elastic modulus E_{tot} is calculated from:

$$D^{3/2} = \frac{k_c \delta}{E_{tot}\sqrt{R}}.$$

Finally, the elastic modulus E is given by:

$$\frac{1}{E_{tot}} = \frac{3}{4}\left(\frac{1-\nu^2}{E} + \frac{1-\nu_t^2}{E_t}\right),$$

where E and ν (E_t and ν_t) are Young's modulus and Poisson's ratio of the sample (of the tip). With E_t = 160 GPa and ν =0.28 for silicon and ν = 0.36 for PMMA, the fit yields E = 9.5 GPa. A detailed discussion of Hertz theory and of the measurement of elastic moduli of polymers through AFM force–distance curves can be found in [1].

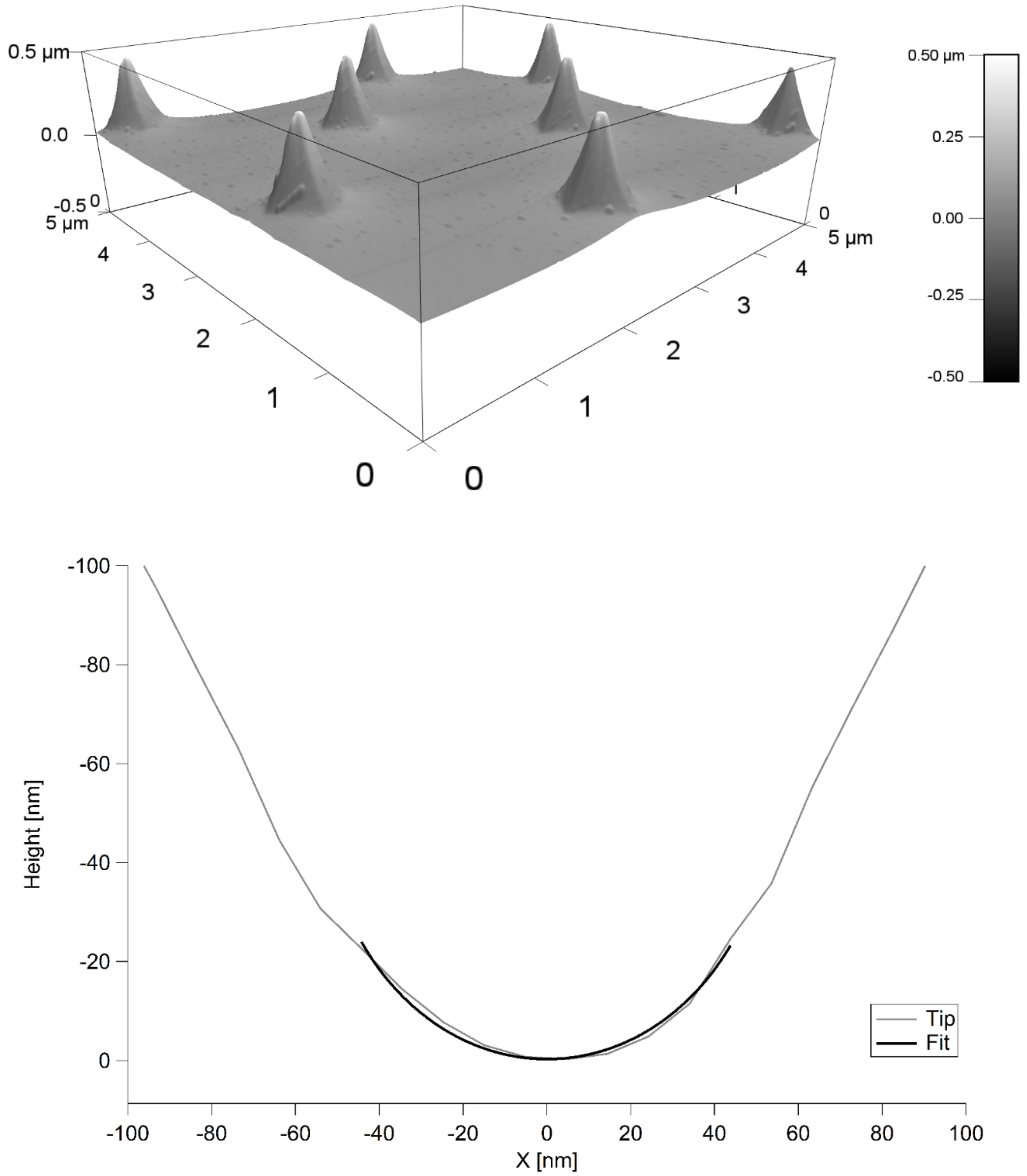

Figure S3: Detection of the tip shape. A TGT1 test grating (NT-MDT Spectrum Instruments, Moscow, Russia) consisting of an array of sharp tips is scanned in tapping mode. The result is an image containing a 3D replica of the tip for each tip of the grid (see top panel). Cross sections are fitted in order to determine the radius of the apex, as exemplarily shown in the bottom panel. The radius is 55 nm. The tip used in this measurement is the same used for the measurements in Figure 5.

References

1. Cappella, B. Mechanical properties of Polymers measured through AFM force-distance curves; Springer: Berlin, Germany, 2016. doi:10.1007/978-3-319-29459-9
